# A Heparan Sulfate-Binding Cell Penetrating Peptide for Tumor Targeting and Migration Inhibition

**DOI:** 10.1155/2015/237969

**Published:** 2015-05-03

**Authors:** Chien-Jung Chen, Kang-Chiao Tsai, Ping-Hsueh Kuo, Pei-Lin Chang, Wen-Ching Wang, Yung-Jen Chuang, Margaret Dah-Tsyr Chang

**Affiliations:** ^1^Institute of Molecular and Cellular Biology, National Tsing Hua University, Hsinchu 30013, Taiwan; ^2^Biomedical Science and Engineering Center, National Tsing Hua University, Hsinchu 30013, Taiwan; ^3^Institute of Bioinformatics and Structural Biology, National Tsing Hua University, Hsinchu 30013, Taiwan; ^4^Department of Medical Science, National Tsing Hua University, Hsinchu 30013, Taiwan

## Abstract

As heparan sulfate proteoglycans (HSPGs) are known as co-receptors to interact with numerous growth factors and then modulate downstream biological activities, overexpression of HS/HSPG on cell surface acts as an increasingly reliable prognostic factor in tumor progression. Cell penetrating peptides (CPPs) are short-chain peptides developed as functionalized vectors for delivery approaches of impermeable agents. On cell surface negatively charged HS provides the initial attachment of basic CPPs by electrostatic interaction, leading to multiple cellular effects. Here a functional peptide (CPP*ecp*) has been identified from critical HS binding region in hRNase3, a unique RNase family member with* in vitro* antitumor activity. In this study we analyze a set of HS-binding CPPs derived from natural proteins including CPP*ecp*. In addition to cellular binding and internalization, CPP*ecp* demonstrated multiple functions including strong binding activity to tumor cell surface with higher HS expression, significant inhibitory effects on cancer cell migration, and suppression of angiogenesis* in vitro* and* in vivo*. Moreover, different from conventional highly basic CPPs, CPP*ecp* facilitated magnetic nanoparticle to selectively target tumor site* in vivo*. Therefore, CPP*ecp* could engage its capacity to be developed as biomaterials for diagnostic imaging agent, therapeutic supplement, or functionalized vector for drug delivery.

## 1. Introduction

Carcinoma is a malignant cancer originating in the ectodermal and endodermal epithelial cells. Interaction between cell surface and microenvironment plays a crucial role in malignant tumor progression. Alterations of cell surface receptor, coreceptor, and adhesive protein expression are reported in various cancer types* in vitro* and* in vivo* [1–3]. Abnormal expression of cell surface molecules notably contributes to enhance tumor cell growth, survival, migration, and invasiveness [4]. Characterization of such alterations and development of novel agent for specific targeting are unmet medical need for early cancer diagnosis.

Glycosaminoglycans (GAGs) including heparan sulfate (HS), chondroitin sulfate (CS), keratan sulfate (KS), or dermatan sulfate (DS) are covalently attached to their core proteins to form proteoglycans. HS proteoglycan (HSPG) present in the extracellular matrix (ECM) provides structural frameworks to mediate cell-cell communication and function in growth factor-receptor binding [5, 6]. HSPGs are key players in modulating tumor progression processes including metastasis, angiogenesis, proliferation, and malignant transformation [4]. Thus, upregulation of cell surface HS may play an active and crucial role in directing malignant phenotype of cancer during different developmental stages.

Cell penetrating peptides (CPPs) are short-chain cationic and/or amphipathic peptides which may be internalized into living cells [7]. CPPs are able to mediate translocation of a conjugated cargo (e.g., anticancer therapeutics) across plasma membrane, providing an effective and nontoxic mechanism for drug delivery [8]. Most CPPs are rich in positively charged Arg and Lys residues and are internalized after initially interacting with cell surface negatively charged GAGs which cluster CPPs on outer membrane surfaces [9, 10].

CPPs might be potentially used in clinical procedures such as gene therapy and cancer therapy [8, 11]. However, most CPPs are unfeasible for* in vivo* researches due to nonspecificity of their highly cationic characteristics. Cell surface negatively charged HS initializes the contact of CPPs, so particular HS binding CPPs might own mysterious sequence to exert multiple functions including HS binding, cellular binding, lipid binding, and* in vivo* tissue targeting activities. CPP*ecp* is a recently identified CPP not only binding to negatively charged molecules including GAGs and lipids on cell surface* in vitro* but also targeting mucosal tissues* in vivo* [12–14]. In this study, we aim to collect and analyze the characteristics of HS-binding cell penetrating peptides derived from natural proteins. Besides, CPP*ecp* itself falling in this classification has demonstrated multiple functions including* in vitro* tumor binding, tumor migration inhibition and angiogenesis inhibition activities, and* in vivo* cargo delivery to tumor site. Here, we provide more clues for the design of peptide therapeutics or intratumor delivery strategy by linking of a tumor targeting CPP. Furthermore, CPP*ecp* might be a unique HS probe for cancer diagnosis to facilitate the quality of therapeutic index and molecular imaging in translational medicine.

## 2. Materials and Methods

### 2.1. Synthetic Peptides

Peptides CPP*ecp* (NYRWRCKNQN) and EDN^32–41^ (NYQRRCKNQN) or CPP*ecp* with* N*-terminally conjugated fluorescein isothiocyanate (FITC) or tetramethylrhodamine (TMR) were synthesized by Genemed Synthesis Inc. and their purities (>90%) were assessed by analytical high-performance liquid chromatography. Peptide sequences were confirmed by matrix-assisted laser desorption/ionisation time-of-flight mass spectrometry in Genemed Synthesis Inc.

### 2.2. Flow Cytometry

Cells (3.0 × 10^5^/well) were added into six-well plates and cultured in the indicated medium. After 24 h, 5 *μ*M FITC-CPP*ecp* dissolved in medium was added into a well and the samples were incubated for 1 h. Cells were then harvested, washed, and suspended in PBS. The fluorescent intensities of the cell samples were measured using a FACSCalibur flow cytometer (BD Biosciences, Franklin Lakes, NJ) and excitation and emission wavelengths of 488 nm and 515–545 nm, respectively. The relative internalization of FITC-CPP*ecp* was reported as the mean fluorescent signal for 10,000 cells.

### 2.3. Fluorescence Microscopy

CT-26 cells were cultured on coverslips (5.0 × 10^3^/coverslip) in RPMI-1640. After 24 h, cell samples were incubated with FITC or FITC-CPP*ecp* at 37°C for 10 min. Alternatively, CT-26 cells were pretreated with heparinase II ( 2.5 mU/mL) (Sigma-Aldrich, Missouri, USA) at 37°C for 2 h followed by treatment with 5 *μ*M TMR-CPP*ecp* at 37°C for 10 min. The cells were then washed twice with PBS, fixed with 4% (w/v) paraformaldehyde, and rinsed twice with PBS. The coverslips were mounted in a Vectashield antifade mounting medium with DAPI (Vector Labs). Inverted fluorescent microscopy was performed using Axiovert 135 (Carl Zeiss, Göttingen, Germany) to assess the distribution of the FITC-CPP*ecp* or TMR-ECP*ecp* in the cells.

### 2.4. *In Vitro* Cell Migration Assay

Effect of CPP*ecp* on cell migration was assessed using a 24-well transwell plate inserted with incorporating polyethylene terephthalate filter membrane with 8 *μ*m pores (BD FalconTM Cell Culture Insert System).

Approximately 4 × 10^4^ CT-26 cells (obtained from ATCC, number: CRL-2638) were suspended in 200 *μ*L of serum-free RPMI-1640 medium (Sigma-Aldrich, Missouri, USA) and pretreated with 1.25, 2.5, 5, and 12.5 *μ*M CPP*ecp* or EDN^32–41^ at RT for 30 min, and then seeded on the upper compartment of transwell insert membrane. The lower compartment of membrane containing 300 *μ*L 1% FBS (Gibco/Invitrogen) RPMI-1640 medium was used as chemoattractant. After incubating at 37°C and 5% CO_2_ for 18 h, the migrated cells on the lower surface of membrane were fixed with 4% formaldehyde for 15 min and stained with 0.05% crystal violet for 20 min. The nonmigrated cells on the upper surface of membrane were removed by cotton swab. Numbers of migrated cells were counted in a randomly selected microscopic field (100x) using inverted microscopy (Olympus CK40, Artisan Technology Group, Mercury Drive Champaign, USA).

Approximately 5 × 10^4^ human umbilical vein endothelial cells (HUVECs) (obtained from BCRC, number: H-UV001) were suspended in 200 *μ*L complete EC medium (Gibco) containing 0, 5, or 12.5 *μ*M CPP*ecp* and then seeded on the upper compartment of filter. The lower compartment of filter contains 500 *μ*L complete EC medium with 20 ng/mL VEGF (R&D) as stimulator. After incubating at 37°C and 5% CO_2_ for 4 h, the migrated cells on the lower surface of filter were fixed with 4% formaldehyde at RT for 15 min and stained with Hocechst at RT for 15 min. The nonmigrated cells on the upper surface of filter were removed by cotton swab. Filter membrane of transwell insert was cut down and mounted with Fluoromount mounting medium (Sigma Aldrich, Missouri, USA). Numbers of migrated cells were counted in five randomly selected microscopic fields at magnification 100x using inverted fluorescent microscope (TE2000E, Nikon, Kanagawa, Japan) with a cooled CCD (Evolution VF, MediaCybernetics, Bethesda, MD).

The result was represented as mean ± SD (standard deviation) of three independent experiments. Statistically significant differences were analyzed using unpaired Student's *t*-test. Asterisks showed level of statistical significance: ^*^ *P* < 0.05; *P* < 0.01; ^***^ *P* < 0.001 compared with control.

### 2.5. Zebrafish Angiogenesis Model

Tg(kdr:EGFP) zebrafish, a well-studied model for vascular embryogenesis [15], was performed to assess the effects of CPP*ecp* on angiogenesis. The Tg(kdr:EGFP) (kindly provided by Dr. Yung-Jen Chuang's lab at NTHU) is a transgenic zebrafish line that expresses eGFP driven by the kdr promoter in vasculature endothelial cells during zebrafish embryogenesis, which can serve as an* in vivo* angiogenesis model for drug screening [16]. Fertilized eggs were generated from adult mating pairs and incubated at 28.5°C in a recirculating aquaculture system. The zebrafish embryos were separately injected with 6.3 or 31.5 ng CPP*ecp* (4.6 nL; 4.56 or 22.8 pmol) into yolk sac at 60 h postfertilization (hpf), and PBS injection was set as control (16–20 zebrafish were used for each treatment condition). After incubating for 24 h, development of subintestinal vessels (SIV) pattern in the zebrafish yolk sac was observed and imaged by inverted fluorescent microscope (TE2000E, Nikon, Kanagawa, Japan) with a cooled CCD (Evolution VF, MediaCybernetics, Bethesda, MD).

### 2.6. Animal Model

All work performed with animals was approved by the Institutional Animal Care and Use Committee at the National Tsing Hua University. Five-week-old female Balb/c mice (supplied by National Laboratory Animal Center, Taiwan) were housed in laboratory animal room at National Tsing Hua University and allowed to adapt to new surrounding for about seven to fourteen days. Animal rooms had a twelve-to-twelve-hour light-dark/day-night cycle and were maintained at constant temperature and humidity. For establishment of tumor-bearing mouse model, CT-26, a mouse colon carcinoma cell was suspended at a density of 1 × 10^6^ cells in 100 *μ*L PBS containing 50% Matrigel (BD Biosciences, San Jose, CA) and subcutaneously injected into the right back of each mouse. Once subcutaneous tumor volumes grew up to 100 mm^3^, all mice were subjected to various treatments. At the end of the experiment, the mice were sacrificed by CO_2_ narcosis. All of the organs including kidney, liver, spleen, trachea, lung, intestine, heart, pancreas, stomach, and tumor of these mice were taken, fixed with paraformaldehyde, embedded in paraffin, and sliced into 5 *μ*m tissue slides for Prussian blue staining.

### 2.7. Magnetic Nanoparticle Conjugated CPP*ecp* and Prussian Blue Staining

To analyze* in vivo* tissue targeting of CPP*ecp*, we have conjugated CPP*ecp* onto a dextran-coated Fe_3_O_4_ type of magnetic nanoparticle (MNP) to form MNP-conjugated CPP*ecp* (MNP-CPP*ecp*) with a mean diameter of 59.3 nm (kindly provided by MagQu. Co., Ltd.) [17]. CT-26 tumor-bearing mouse was utilized to investigate biodistribution of MNP-CPP*ecp* and Prussian blue staining was employed to demonstrate ferric iron in mouse tissues. The CT-26 tumor-bearing mouse was intravenously injected with 150 *μ*L MNP-CPP*ecp* (0.06 emu/g) and sacrificed by CO_2_ narcosis at a time point of 3, 6, 12, and 24 h after administration. The kidney, heart, liver, spleen, stomach, pancreas, small intestine, large intestine, trachea, lung, and tumor of mice were taken, fixed with paraformaldehyde, embedded in paraffin, and sectioned into 5 *μ*m thick tissue slides, following by deparaffinizing in xylene solution (J. T. Baker Phillipsburg, NJ, USA) and serially rehydrating with 100%, 95%, 85%, 75%, and 50% alcohol. The slides were continuously immersed in working solution (20% hydrochloric acid and 10% potassium ferrocyanide (Sigma, MO, USA) solution mixture, 1 : 1 volume ratio) at room temperature for 30 min and then counterstained with fast nuclear red (Sigma, MO, USA) at RT for 5 min. After dehydration through 95% and 100% alcohol and clearing with xylene, each slide was finally covered with coverslip. Tissue images were digitized using light microscope (Eclipse E400, Nikon) with digital microscopy camera (AxioCam ICc 5, ZEISS).

## 3. Results and Discussion

### 3.1. Heparan Sulfate Binding Cell Penetrating Peptides Derived from Natural Proteins

Heparan sulfate (HS) serves as the initial anchoring site for many CPPs through electrostatic interactions between negatively charged sulfates or carboxyl groups and basic amino acids Arg as well as Lys [18]. Till now 27 CPPs from natural proteins including 14 viral protein-derived peptides, 7 animal homeostatic modulator-derived peptides, 3 antimicrobial peptides, and 3 toxin-derived peptides have been demonstrated or predicted to be able to interact with cell surface HS and penetrate cross the plasma membrane.* In silico* secondary structures of all 27 HS-binding CPPs were predicted by Network Protein Sequence Analysis [19]. As shown in Table 1, 17 peptides including CPPs 2–6, 8–12, 15, and 18–23 exist as *α* helix (H). Seven peptides including CPPs 1, 7, 13, 14, 16, 17, and 24 form random coil (C). CPP 23 exists as *β* sheet (E), and CPPs 26 and 27 exist as mixed *α* helix (H) with *β* sheet (E) structures. Among 27 CPPs seventeen structures have been validated by* in vitro* 3D structures deposited in Protein Data Bank (Table 1, underline) [20]. All 14 viral protein-derived CPPs are highly cationic (high pI values) with 10 peptides forming *α* helix and 4 existing as random coil, penetrating cells through direct translocation [21–24] and lipid raft-mediated endocytosis [25–29]. Most of the 7 animal homeostatic modulator-derived CPPs may be internalized into cytosol through HS-mediated and energy-dependent endocytosis, among which 5 animal protein-derived peptides are demonstrated to possess either *α* helix or *β* sheet to interact with the plasma membrane, while our CPP*ecp* and apolipoprotein B binding domain are unique such that they hold random coil structures in this category. As for 3 antimicrobial peptides, all of them are suggested to interact with cell surface HS and penetrate membrane barrier via energy-dependent endocytosis. LL-37 holds high level of *α* helix, SynB1 possesses *β* sheet, and SynB3 retains random coil structures [30–32]. For the last category toxin-derived CPPs, bovine prion-derived bPrPp forming *α* helix and mixed *α* helix with *β* strand are distributed in the internal region of venom-derived crotamine, and scorpion toxin-derived maurocalcine [33–36].

Previous researches have shown that the interactions between the positively charged peptide and highly negatively charged membrane components, such as the GAG moieties of cell surface proteoglycans, play a crucial role in the overall process of cellular permeability of highly basic or amphipathic CPPs [37]. Although this investigation may also reflect nonspecific electrostatic interactions between these basic peptides and HS, it has been characterized that negatively charged heparin more effectively blocks uptake of CPPs than other soluble GAGs such as CS and hyaluronic acid [38], likely suggesting that there might be some structural requirements involved in the strong interaction between CPP and HS. In Table 1, 19 of these 27 HS-binding CPPs generally possess conventional heparin binding sequences such as XBBXB and XBBBXXBX where B is a basic amino acid and X represents a random amino acid, and they can also be divided into cationic and amphipathic groups. Most viral factor-derived peptides are basic amino acid-rich. For example, cationic TAT is an extensively used CPP rich in Arg and can interact with sulfated proteoglycans and negatively charged phospholipids on the cell membrane [25]. It should be noted that although 10-amino acid CPP*ecp* is almost equal size to 9-residue TAT and 10-residue SynB3, the features of TAT and SynB3 are quite different from CPP*ecp*. Both TAT derived from viral protein and SynB3 belonging to antimicrobial peptide are highly cationic peptides with high pI values above 12, while our newly identified CPP*ecp* containing only 2 Arg and 1 Lys in a total of 10 amino acids is amphipathic with a pI value of 10.05. Interestingly, the proportion of basic residues in amphipathic crotamine (26%) is close to CPP*ecp* (30%). “RWRCK” motif of CPP*ecp* was previously predicted as a unique functional pattern in all 13 hRNaseA family members employing Reinforced Merging for Unique Segments system (ReMUS) [39]. Another peptide CyLoP-1 (CRWRWKCCKK) derived from crotamine also exhibited efficient intracellular delivery activity. In both cases positively charged residues conducting electrostatic interaction and aromatic Trp exerting transient membrane destabilization were essential to maintain CPP functionality [40, 41]. Taken together, a similar motif  “RWRXK” shown on the loop, where X might be a random amino acid, is present in both CPP*ecp* and crotamine, suggesting that combination of positively charged residues and nonpolar aromatic residues, especially Trp, might provide a design rationale for novel amphipathic cell penetrating peptides.

### 3.2. Cellular Binding of CPP*ecp* to Tumor Cell with Higher HS Expression Level 

**Figure 1:**
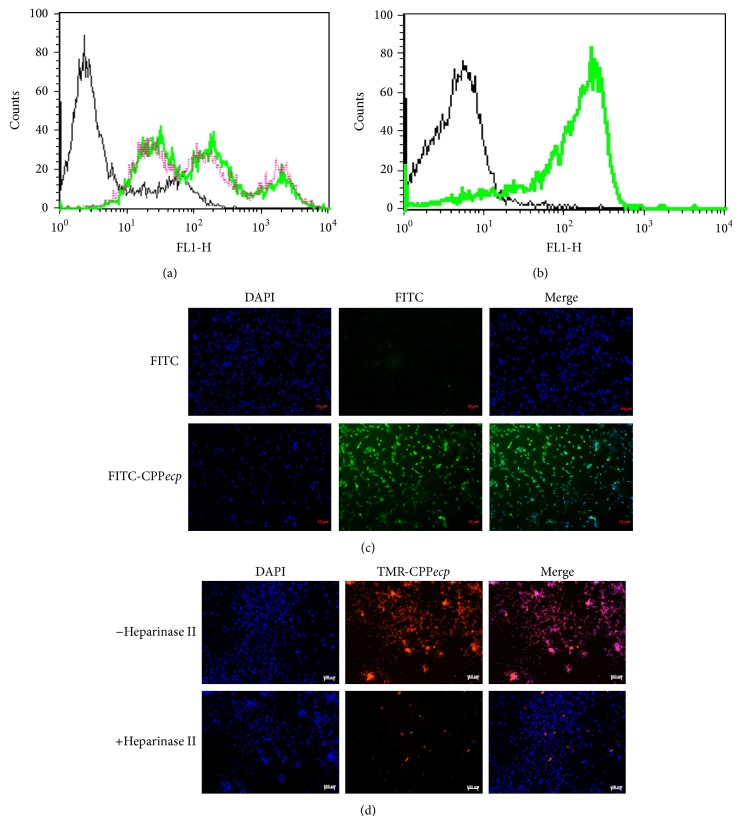
Effect of surface HS level on CPP*ecp* binding to CT-26 cells. (a) CT-26 cells were preincubated at 4°C for 30 min and then incubated with 5 *μ*M FITC-CPP*ecp* for 1 h. The cells were washed twice with 500 *μ*L PBS, trypsinized at 37°C for 15 min, suspended in 500 *μ*L PBS, and subjected to flow cytometry. (b) CT-26 cells were stained with anti-HS monoclonal antibody (10E4) at 4°C for 1 h, washed twice with 500 *μ*L PBS, and hybridized with FITC-conjugated anti-mouse secondary antibody at 4°C for 1 h. After being washed twice with 500 *μ*L PBS, cells were suspended in 500 *μ*L PBS and subjected to flow cytometry. (c) CT-26 cells were treated with 5 *μ*M FITC-CPP*ecp* at 37°C for 10 min. Uptake of FITC-CPP*ecp* by CT-26 cells was examined by fluorescent microscopy. FITC was set as a negative control. DAPI staining of cells indicated intact nucleus. Scale bars in panel represented 10 *μ*m. Green, FITC-labeled CPP*ecp*; blue, DAPI (nucleus). (d) CT-26 cells were pretreated with or without heparinase II (2.5 milliunit/mL) at 37°C for 2 h followed by treatment with 5 *μ*M TMR-CPP*ecp* at 37°C for 10 min. Uptake of TMR-CPP*ecp* by CT-26 cells was examined by fluorescence microscopy. TMR-CPP*ecp* bound on CT-26 tumor cell DAPI staining of cells indicated intact nucleus. Scale bars in panel represented 10 *μ*m. Red, TMR-labeled CPP*ecp*; blue, DAPI (nucleus).

Heparan sulfate (HS) is reported to be overexpressed in several tumors [42, 43], while HSPG profiles on different tumor cell surface are largely unclear. Here a mouse colon cancer CT-26 cell line was used for* in vitro* and* in vivo* analyses. Cellular binding activity of CPP*ecp* and HS expression level on cell surface of CT-26 cells were accessed for quantitative analysis employing flow cytometry and fluorescent microscopy with fluorescence-labeled CPP*ecp* FITC-CPP*ecp* and an anti-HS monoclonal antibody recognizing an epitope of* N*-sulfated glucosamine on membrane HS (US Biological, Swampscott, MA, USA).  Figure 1(a) showed significant FITC-CPP*ecp* binding activity to CT-26 cells, which correlated well with significantly higher HS expression (Figure 1(b)). In addition, 5 *μ*M FITC-CPP*ecp* rapidly and efficiently internalized into CT-26 cells within 10 min as analyzed by fluorescent microscopy (Figure 1(c)). To further address the importance of HS for CPP*ecp* anchor in the absence of autofluorescence background, removal of cell surface HS by heparinase was carried out along with CPP*ecp* labeled with tetramethylrhodamine (TMR). CT-26 cells were incubated in medium with (+) or without (−) heparinase II for 2 h and then treated with 5 *μ*M TMR-CPP*ecp* for 10 min. TMR-CPP*ecp* rapidly and efficiently bound to CT-26 cell surface (Figure 1(d), upper panel), while removal of cellular HS led to significant reduction in CPP*ecp* attachment (Figure 1(d), lower panel). Taken together, our HS-binding CPP*ecp* possessed strong binding activity to tumor cell surface with higher HS expression, while depletion of cell surface HS abolished such highly selective binding activity of CPP*ecp* to tumor cells.

### 3.3. Effect of CPP*ecp* on Migration of Mouse Colon Carcinoma Cell

**Figure 2:**
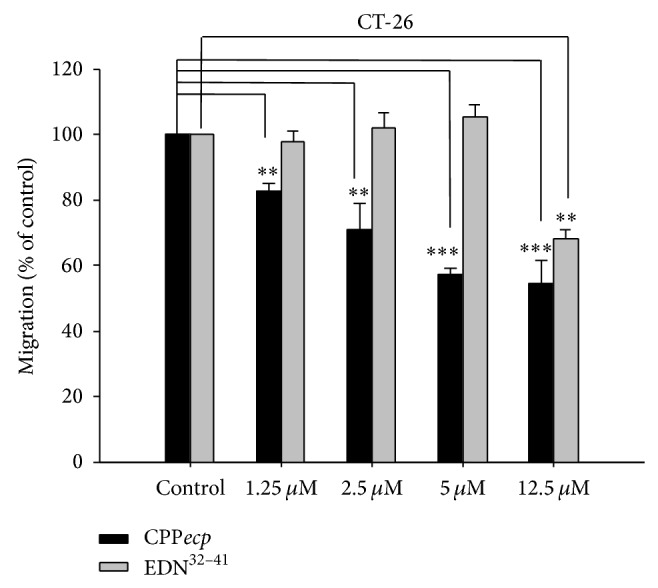
Inhibitory effect of CPP*ecp* on CT-26 cell migration. CT-26 cells were pretreated with CPP*ecp* or EDN^32–41^ at indicated concentration in serum-free RPMI-1640 medium at room temperature for 30 min and then seeded onto the upper side of transwell insert membrane at 37°C for 18 h. Number of migrated cells without CPP*ecp* or EDN^32–41^ treatment was set as 100%. The data represents means ± SD (standard deviation) of three independent experiments. ^*^ *P* < 0.05; ^**^ *P* < 0.01; ^***^ *P* < 0.001 compared with control.

It has been shown that HSPGs may modulate cell migration by interacting with growth factors or chemokines and drives cell migrate toward specific stimuli [44]. Since CPP*ecp* with a novel heparin-binding motif in ECP has already been identified to possess high recognition activity to cellular surface HSPG and penetration activity into cells [12], here whether CPP*ecp* might modulate cancer cell migration through interaction with HSPG was further investigated using* in vitro* transwell migration assay, while EDN^32–41^, a 10-amino acid peptide derived from comparable sequence motif of human RNase2 (EDN), possessing a conventional heparin-binding motif was also analyzed as a control.  Figure 2 (black bar) showed that migration activity of CT-26 cell was significantly inhibited by CPP*ecp* in a dose-dependent manner such that it decreased to 83%, 71%, 56%, and 54% upon treatment with 1.25, 2.5, 5, and 12.5 *μ*M CPP*ecp*, respectively. Yet treatment with 1.25, 2.5 and 5 *μ*M EDN^32–41^ could not inhibit migration activity of CT-26 cells, and presence of higher concentration of EDN^32–41^ (12.5 *μ*M) decreased 33% tumor migration (Figure 2, gray bar). These results indicated that CPP*ecp* containing core RWRCK motif, rather than containing known heparin-binding motif, inhibited CT-26 cell migration across the membrane* in vitro*. It has been reported that cancer migration was inhibited by antagonism of HS side chains. For example, A5G27 peptide derived from laminin *α*5 globular domain recognizes HS side-chains of CD44 variant 3 and blocks bioactivity of fibroblast growth factor-2 (FGF-2). It significantly inhibits FGF-2-induced WiDr colon cancer cell migration and invasion [45]. Collectively, inhibitory effect of CPP*ecp* on cancer cell migration is possibly arisen from interaction with cell surface HS.

### 3.4. Effects of CPP*ecp* on Migration of Vascular Endothelial Cell

**Figure 3:**
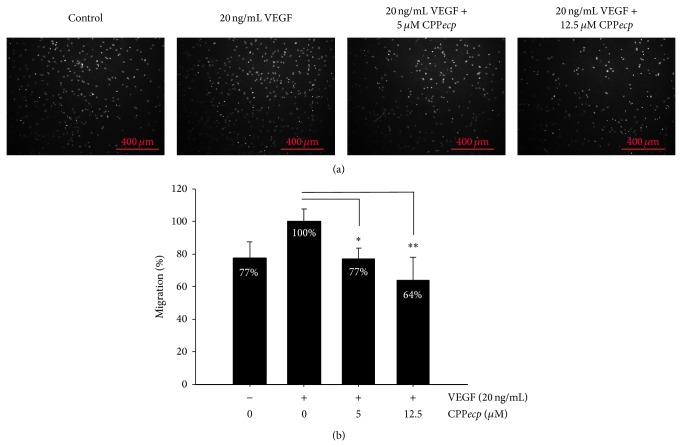
Inhibitory effect of CPP*ecp* on HUVEC migration. HUVECs were seeded onto the upper side of transwell insert membrane containing CPP*ecp* at indicated concentration at 37°C for 4 h. The lower side of transwell was filled with complete EC medium supplementing with 20 ng/mL VEGF. Migrated cells on the lower surface of transwell insert membrane were stained with Hoechst (a). Percentage of migrated cells in the presence of VEGF and set as 100% (positive control). Alternation of HUVEC migration activity in the presence of VEGF and various concentrations of CPP*ecp* were quantified as compared with positive control (b). The data represents means ± SD (standard deviation) of three independent experiments. ^*^ *P* < 0.05; ^**^ *P* < 0.01; ^***^ *P* < 0.001 compared with control. Magnification: 100x. Scale bar: 400 *μ*m.

Cell surface HS proteoglycan (HSPG) serves as a coreceptor to coordinate binding of vascular endothelial growth factor (VEGF) toward its receptor. It has been reported to be associated with angiogenesis [46, 47]. However, vascular endothelial cell migration is a crucial step in formation of new blood vessel and tumor angiogenesis [48]. To test the hypothesis that CPP*ecp* interacting with cell surface HSPGs also affected angiogenesis, a common model cell line human umbilical vein endothelial cell (HUVEC) was used for* in vitro* transwell migration assay.  Figure 3 indicated that VEGF-induced HUVEC migration was restored by cotreatment with 5 or 12.5 *μ*M CPP*ecp*, leading to, respectively, 77% and 64% migration activity. This result indicated that CPP*ecp* could inhibit VEGF-induced HUVEC migration. Likewise, the CD44-binding peptide A5G27 derived from laminin *α*5 globular domain inhibits FGF-induced angiogenesis in Chick CAM Assay [49]. Moreover, an HS-binding peptide 6a-P, corresponding to the HSPG binding domain of VEGF, binds to HSPG and affects interaction between VEGF and HSPG [50]. It interferes with angiogenesis by inhibiting VEGF-induced HUVEC migration and binding of VEGF to HUVEC. As a result, involvement of our CPP*ecp* in angiogenesis may be attributed to interaction with cell surface HSPG.

### 3.5. Effects of CPP*ecp* on Angiogenesis during Embryonic Development of Zebrafish

**Figure 4:**
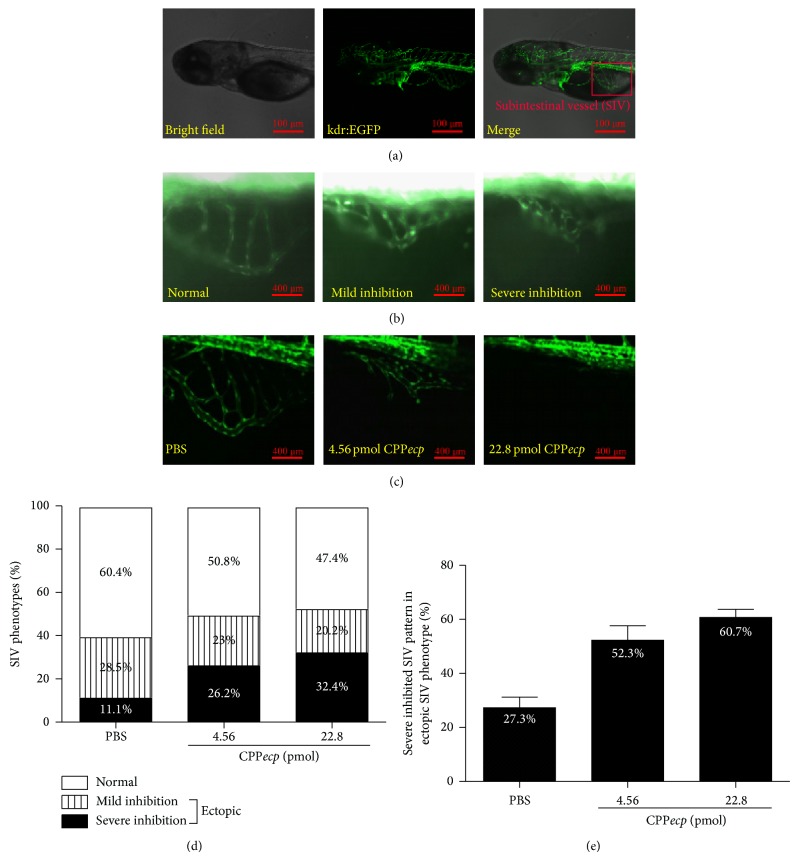
Inhibitory effect of CPP*ecp* on angiogenesis in Tg(kdr:EGFP) zebrafish. (a) Morphology and green-labeled vessels in Tg(kdr:EGFP) zebrafish. The red rectangle represents the area of subintestinal vessel (SIV) network. Magnification: 100x. Scale bar: 400 *μ*m. (b) Development of SIV network in the zebrafish yolk sac could be classified into three groups: normal, mild inhibition, and severe inhibition pattern. Magnification: 400x. Scale bar: 100 *μ*m. (c) Development of SIV network in the zebrafish yolk sac at 24 h postinjection (hpi). Magnification: 400x. Scale bar: 100 *μ*m. (d) Percentage of different SIV phenotypes in the zebrafish yolk sac at 24 hpi. (e) Percentage of severe inhibited SIV phenotype in ectopic SIV phenotype in the zebrafish yolk sac at 24 hpi. 16–20 zebrafish were used for each treatment group. The data represents means ± SD (standard deviation) of three independent experiments.

Cell surface HSPGs serve as a coreceptor to coordinate binding of VEGF toward its receptor and have been reported to be associated with angiogenesis [46, 47]. Tg(kdr:EGFP) zebrafish, a well-studied model for vascular embryogenesis, has been used as model for drug screening and angiogenesis studies [51, 52]. It was thus utilized to investigate CPP*ecp* effects on* in vivo* embryonic angiogenesis by injecting 4.6 nL of 4.56 or 22.8 pmol CPP*ecp* or PBS (control) into yolk sac of zebrafish at 60 h postfertilization (hpf), and the development of subintestinal vessel (SIV) pattern (Figure 4(a), SIV networks are indicated with red rectangle) at 24 h postinjection (hpi) was monitored with images by inverted fluorescent microscope. Here 16–20 zebrafish were tested for each treatment group. The observed SIV patterns of zebrafish were divided into three groups according to growth level of SIV: normal, mildly inhibited, and severely inhibited phenotypes (Figure 4(b)). In the normal phenotype SIV developed as smooth basket-like pattern with 5-6 arcades. Both mild and severe inhibition phenotypes could be further classified as ectopic SIV pattern, in which SIV exhibited tortuous network and was unable to demonstrate complete basket-like pattern that normal phenotype developed. However, severe inhibition phenotype displayed more incomplete SIV network than mild inhibition phenotype did. In contrast, the zebrafish injected with CPP*ecp* appeared to be tortuous, in which SIV pattern shrank significantly as compared with that of PBS control (Figure 4(c)).  Figure 4(d) illustrated quantitative analysis data in which percentage of ectopic SIV phenotype (mildly inhibited phenotype plus severely inhibited phenotype) rose from 39.6% up to 49.2% and 52.6% upon injection with 4.56 and 22.8 pmol CPP*ecp*, respectively. Moreover, severely inhibited SIV phenotype increased from 11.1% up to 26.2% and 32.4% upon injection with 4.56 and 22.8 pmol, respectively. In other words, percentage of severely inhibited phenotype in ectopic phenotype of zebrafish increased from 27.3% (control) up to 52.3% and 60.7% upon injection with 4.56 and 22.8 pmol CPP*ecp*, respectively (Figure 4(e)). These data revealed that our CPP*ecp* possessed antiangiogenesis activity in inhibiting SIV growth of zebrafish. As a result, involvement of CPP*ecp* in angiogenesis may be attributed to interaction with cell surface HS. CPP*ecp* is the first antiangiogenic peptide deciphered in embryonic development of zebrafish.

### 3.6. Time-Dependent Biodistribution of MNP-CPP*ecp* in CT-26 Tumor-Bearing Mouse

**Figure 5:**
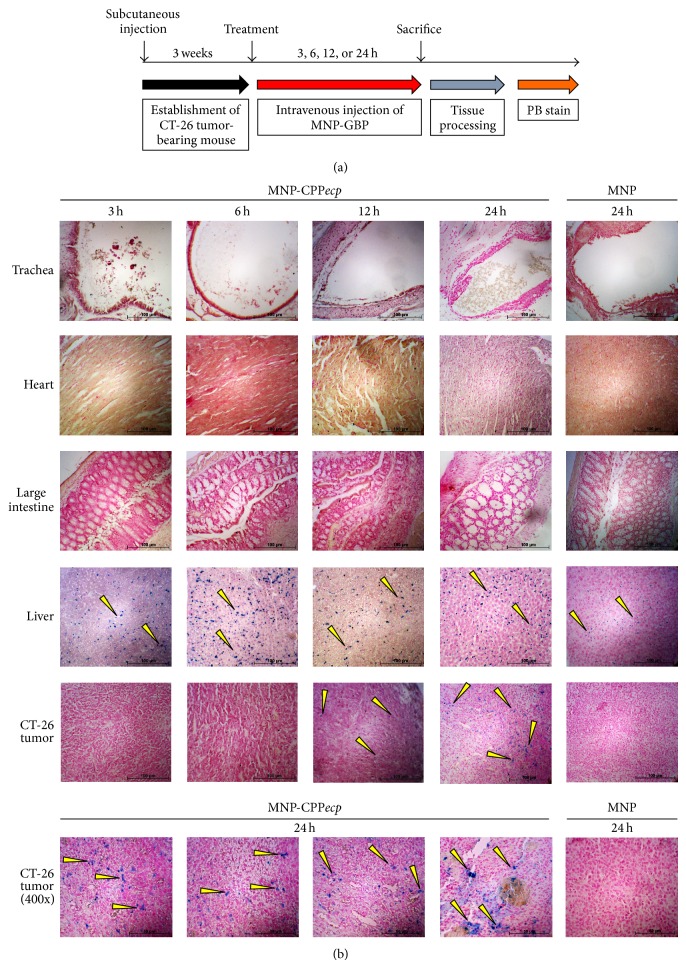
Localization of MNP-CPP*ecp* in CT-26 tumor-bearing mouse. To investigate the biodistribution of CPP*ecp in vivo*, CT-26 tumor-bearing mice were intravenously injected with 0.06 emu/g MNP-CPP*ecp* and sacrificed at a time point of 3, 6, 12, and 24 h after injection (a). Signal of MNP-CPP*ecp* was visualized using Prussian blue staining to indicate ferric iron in tissue section (blue color, yellow arrow). Represented staining patterns of trachea, heart, large intestine, liver, and CT-26 tumor were shown (b). MNP injection was set as negative control. Nuclear fast red staining was set as counterstain (red color). Magnification: 200x and 400x. Scale bar: 100 *μ*m and 50 *μ*m.

To better understand biodistribution of our HS-binding CPP*ecp in vivo*, CPP*ecp* was conjugated with well-dispersed Fe_3_O_4_ magnetic beads (59.3 nm for diameter) to form magnetic nanoparticle-conjugated CPP*ecp* (MNP-CPP*ecp*). CT-26 tumor-bearing mice were intravenously injected with MNP-CPP*ecp* (0.06 emu/g) and sacrificed at different time point after administration (Figure 5(a)). MNP-CPP*ecp* signal was detected using Prussian blue staining to indicate ferric iron in tissue section (blue color).  Figure 5(b) indicated that stainable ferric iron (blue color as indicated by yellow arrow) was barely detectable in trachea, heart, and large intestine at all indicated time points, and so did other tissues including stomach, pancreas and kidney (data not shown). The MNP-CPP*ecp* mainly accumulated in liver tissues from 3 h up to 24 h owing to uptake and removal by macrophages of reticuloendothelial system, which played a role in clearance of external substance in liver [53, 54]. Interestingly, Prussian blue staining signals in CT-26 tumor section suggested MNP-CPP*ecp* accumulation from 12 h to 24 h, whereas MNP signal was only detected in liver at 24 h. One recent report showed that exendin-4 peptide-conjugated superparamagnetic iron oxide nanoparticles were inevitably accumulated in liver tissue, suggesting that a nanoparticle might unavoidably be captured by this metabolic organ [55]. However, it is worth noting that CPP*ecp* has potential to target colon carcinoma* in vivo*, suggesting that CPP*ecp* might be applied for a potent carrier for drug delivery.

### 3.7. Heparan Sulfate-Binding Cell Penetrating Peptide for Tumor Targeted Strategy

Although CPPs as noninvasive agents have promising biomedical potential for molecular delivery, they are mostly unfeasible for* in vivo* researches due to nonspecificity of their highly cationic characteristic such as TAT peptide. Due to high uptake rates* in vitro* and relatively low specificity* in vivo* of most CPPs, conventional CPPs would be designed for topical applications in CPP-based delivery (Table 1). Further analysis of natural protein-derived CPPs revealed that 5 CPPs exerted* in vitro* tumor suppression as well as cell internalization activities (Table 2). Although TAT peptide (46–57) demonstrated antiangiogenesis and apoptosis-inducing activities, TAT peptide was proved to show low target specificity* in vivo* [56]. Distinct from conventional highly cationic CPPs, 4 amphipathic CPPs including CPP*ecp*, crotamine, NFL-TBS (40–63), and p28 peptides demonstrated unique tumor targeting activity* in vivo*. Even though specific protein receptors for CPP*ecp* and crotamine remain to be investigated, HSPG acting as coreceptor is indispensable for the translocation of CPP*ecp* and crotamine [12, 34]. In addition, both CPP*ecp* and crotamine targeted highly proliferating cells such as tumor tissues [14, 57]. Interestingly, a motif decorating a hydrophilic aromatic amino acid participating in membrane permeation between two arginines (RWR) appeared to be conserved in both CPP*ecp* and crotamine, leading to similar characteristics of these 2 multifunctional HS-binding CPPs. Therefore, amphipathic CPPs might own promising potential to be designed as peptide-based drugs. In particular, HS-binding CPPs are suitable drug carriers for* in vivo* application in delivery of functional therapeutics.

## 4. Conclusions

CT-26 colon tumor cells revealed high CPP*ecp* binding activity due to high HSPG expression on cell surface. CPP*ecp* displays not only significantly inhibitory effects on CT-26 cancer migration and angiogenesis* in vitro* but also antiangiogenesis activity during zebrafish embryogenesis* in vivo*. Moreover, covalent linkage of CPP*ecp* to magnetic nanoparticle shows potential for* in vivo* targeting to a subcutaneous CT-26 tumor site. Moreover, CPP*ecp* containing a core RWRXK sequence demonstrates both cell penetrating and epithelial tumor targeting activities. Taken together, our HS-binding CPP*ecp* might be feasible for further application in molecular imaging for tumor homing and selectively targeting drug delivery system.

## Figures and Tables

**Table 1:** pI, sequence, and structures of HS-binding cell penetrating peptides.

	Peptide name pI	Sequence and predicted secondary structure^*^	Heparan sulfate binding region	Internalization mechanism	Ref.
	Viral protein-derived CPP				
1	TAT peptide (49–57) pI: 12.70	**RKKRRQRRR** CCCCCCCCC	RKKRRQRR	Lipid raft-mediated macropinocytosis	[25, 26]
2	Nucleoplasmin NLS (155–170) pI: 11.47	**KRPAAIKKAGQAKKKK** CcHHHHHHHhHHHhCC	Not reported	Not reported	[58]
3	HTLV-II Rex (4–16) pI: 12.85	**TRRQRTRRARRNR** CCCCHHHHCCCCC	TRRQRT	Direct translocation	[21, 22]
4	Lambda-N (48–62) pI: 11.83	**QTRRRERRAEKQAQW** CCHHHHHHHHHHCCC	RRRERR	Not reported	[22]
5	Phi21 N (12–29) pI: 11.45	**TAKTRYKARRAELIAERR** CCCCCCCHHHHHHHHHHH	KTRYKARRA	Not reported	[22]
6	Delta N (1–22) pI: 11.44	**MDAQTRRRERRAEKQAQWKAAN** CCCCHHHHHHHHHHHHHHHHHH	TRRRERRA	Not reported	[22]
7	FHV coat (35–49) pI: 13.00	**RRRRNRTRRNRRRVR** CCCCCCCCCCCCCCC	RRRRNRTRRNRRRVR	Not reported	
8	BMV coat (8–26) pI: 12.78	**KMTRAQRRAAARRNRWTAR** CcCHHHHHHHHHHhccccC	ARRNRW	Not reported	
9	HIV-1 Rev (35–46) pI: 12.85	**RQARRNRRRRWR** CCCCCCCHHHHH	RQARRNRRRRWR	Not reported	[22]
10	Rev (26–42) pI: 12.54	**TRQARRNRRRRWRERQF** CCCCCCCCHHHHHHHHH	TRQARRNRRRRWRERQF	Energy dependent lipid raft-mediated macropinocytosis	[27, 28]
11	CPP from pestivirus envelope glycoprotein (Erns) (194–220) pI: 11.72	**ENARQGAARVTSWLGRQLRIAGKRLEGRSKTWFGAYA** CCCccchHHHHHHHHHHHHHHHHhhhCCCCccccccC	Basic residues	Direct translocation	[23]
12	gp41 fusion sequence pI: 11.33	**GALFLGWLGAAGSTMGAWSQPKKKRKV** HHHHHHHHHHHHHHHHHCCCCCCCCCC	WSQPKKKRKV	Direct translocation	[24]
13	VP22 pI: 12.10	**DAATATRGRSAASRPTERPRAPARSASRPRRPVD** CCCccCCCCCCCCCCCCCCCCCCCCCCCCCCCCC	SRPRRP	Energy dependent lipid raft-mediated macropinocytosis	[27, 29]
14	SV40 NLS pI: 11.33	**PKKKRKV** CCCCCCC	PKKKRKV	Not reported	[59, 60]

	Animal homeostatic modulator-derived CPP				
15	Penetratin pI: 12.31	**RQIKIWFQNRRMKWKK** CCCHHHHHHHCCCCCC	NRRMKW	Direct translocation Endocytosis	[61]
16	CPP*ecp* pI: 10.05	**NYRWRCKNQN** CCCCCCCCCC	RWRCK	Macropinocytosis	[12, 62]
17	Apolipoprotein B binding domain pI: 9.82	**SVKAQYKKNSDKHRLMRKRGLK** CCcccccCCCCCCCCCCcCCCC	Basic residues	Endocytosis	[63, 64]
18	hCT (9~32) pI: 6.74	**LGTYTQDFNKFHTFPQTAIGVGAP** HHHHHHHHHHHHHHCHHHHHCCCC	Not reported	Endocytosis	[63, 65]
19	pVEC (615–632) pI: 12.48	**LLIILRRRIRKQAHAHSK** ChhHHHHHHHHHHHhcCC	LRRRIRK	Macropinocytosis and clathrin mediated endocytosis	[66–68]
20	hLF peptide pI: 10.93	**KCFQWQRNMRKVRGPPVSCIKR** CCCchhHHHHhCCCCCcececC	MRKVRG	Lipid raft-mediated endocytosis	[69]
21	PDX-1-PTD pI: 12.31	**RHIKIWFQNRRMKWKK** ChhhhHhhhhhhhhcC	NRRMKWKK	Caveolae-dependent endocytosis and lipid raft-mediated macropinocytosis	[70]

	Antimicrobial peptide				
22	LL-37 (1–37) pI: 10.61	**LLGDFFRKSKEKIGKEFKRIVQRIKDFLRNLVPRTES** HHHHHHHHHHHHHHHHHHHHHHHHHHHHHHHHCCCCC	FRKSKEKI	Endocytosis	[30, 31, 71]
23	SynB1 (1–18) pI: 12.30	**RGGRLSYSRRRFSTSTGR** CCCCEEEEECCEEEEECC	Basic residues	Endocytosis	[32]
24	SynB3 pI: 12.18	**RRLSYSRRRF** CCCCcccCCC	Basic residues	Endocytosis	[32]

	Toxin-derived CPP				
25	bPrPp (1–28) pI: 10.03	**MVKSKIGSWILVLFVAMWSDVGLCKKRP** CCCCCCCCHHHHHHHHHHHHHHHCCCC	Basic residues	Macropinocytosis	[33]
26	Crotamine (1–42) pI: 9.51	**YKQCHKKGGHCFPKEKICLPPSSDFGKMDCRWRWKCCKKGSG** CCHHHHHCEEEECCCCCCCCCCCEECCCCCCCCCEEEECCCC	RWRWK	Endocytosis	[34]
27	Maurocalcine (MCa) (1–33) pI: 9.46	**GDCLPHLKLCKENKDCCSKKCKRRGTNIEKRCR** CCCCCCCCCCCCHHHCCCCEEECCCCCCCCEEE	SKKCKR and EKRCR	Macropinocytosis	[35, 36]

^∗^The confidence of the prediction is denoted by scaling the predictions from week (lower-case letter) to strong (upper-case letter). “H,” “E,” and “C” refer to *α*-helical, *β*-strand, and random coil propensities, respectively.

**Table 2:** Multifunctional CPPs for tumor suppression.

Name/sequence	Function	Mechanism	Cell line	Tumor mouse model	Ref.
CPP*ecp*/**NYRWRCKNQN**	Cell penetrating HS binding Antimigration Antiangiogenesis Tumor targeting	Block putative HS coreceptor for growth factor	CT-26 HUVEC	Murine colon carcinoma CT-26	[12–14]

Crotamine/**YKQCHKKGGHCFPKEKICLPPSSDFGKMDCRWRWKCCKKGSG**	Cell penetrating HS binding Antiproliferation Tumor targeting	Interact with lysosomes to trigger intracellular Ca^2+^ transients and alter mitochondrial membrane potential	B16F10 CHO-K1	Murine melanoma (B16F10) Murine mammary carcinoma (TS/A-pc, TS/A-pc-pGL3)	[34, 57]

NFL-TBS. (40–63)/**YSSYSAPVSSSLSVRRSYSSSSGS**	Cell penetrating Antimigration Antiproliferation Apoptosis-inducing Antitumor growth	Inhibit polymerization of microtubules	Human glioblastoma (T98G) Rat glioblastoma (F98) Rat gliosarcoma (9L)	Murine glioblastoma (F98)	[72, 73]

TAT peptide (46–57)/**SYGRKKRRQRRR**	Cell penetrating HS binding Antiangiogenesis Apoptosis-inducing	Inhibit VEGF binding to HUVEC and inhibit phosphorylation of ERK	HUVEC	×	[25, 74]

p28/**LSTAADMQGVVTDGMASGLDKDYLKPDD**	Cell penetrating Antiangiogenesis Antitumor growth	Inhibit phosphorylation of VEGFR-2, FAK, and Akt	HUVEC	Human melanoma (UISO-Mel-6)	[75]
